# Factors Associated With Complications in Patients With Hematological Malignancies and Febrile Neutropenia: A Cohort Study

**DOI:** 10.7759/cureus.81750

**Published:** 2025-04-05

**Authors:** Jose C Alvarez-Payares, Santiago Alvarez-Lopez, Jose E. Agámez-Gomez, Juan C. Hernandez-Rodriguez, Alejandra Ramírez-Roldán, Ángel D. Molina-Prado, Manuela Cardona-Jaramillo, Adriana M. Trejos-Tenorio, Sigifredo Ospina-Ospina, Ioka de la Peña-Lozano, Daniel Barrera-Correa, Daniel A. Ribero-Vargas, Edwin J Ariza-Parra, Amado J. Karduss-Urueta

**Affiliations:** 1 Hematology, University of Antioquia, Medellin, COL; 2 Internal Medicine, National University of Colombia, Bogotá, COL; 3 Internal Medicine, University of Antioquia, Medellin, COL; 4 General Medicine, University of Antioquia, Medellin, COL; 5 Internal Medicine, Quiron Salud Hospital, Torrevieja, ESP; 6 Epidemiology, University of Antioquia, Medellin, COL; 7 General Medicine, Pablo Tobón Uribe Hospital, Medellin, COL; 8 Hematology, Bone Marrow Transplant Program, Cancer Institute, Las Americas Clinic, Medellin, COL

**Keywords:** antineoplastic agents, chemotherapy-induced febrile neutropenia, febrile neutropenia, hematological malignancies, infections, mortality

## Abstract

Introduction

Febrile neutropenia (FN) in patients with hematological malignancy (HM) is associated with multiple hospital complications including mortality. Although different strategies for early detection and prompt treatment have been established, it is a heterogeneous population with risk factors that are difficult to detect. The data available on the prediction of such complications is limited and there lies the importance of characterizing this type of patients in our environment and evaluating the factors related to the adverse outcomes.

Methods

The study is a retrospective cohort study conducted at San Vicente Foundation University Hospital (HUSVF) and Alma Mater Hospital of Antioquia (HAMA) in Medellín, Colombia, between January 2018 and December 2020, including patients diagnosed with FN who presented FN at the time of diagnosis or up to 30 days after receiving chemotherapy. The main objective was to determine the factors related to mortality and severe complications (ICU admission, need for vasopressors, or need for mechanical ventilation), while the secondary objective was the microbiological characterization of this population.

Results

Of the 190 FN episodes, 134 (70.5%) had a clinical focus of infection. A causal agent was identified in 125 episodes (65.8%), with the majority being bacteria in 112 cases (92.6%) of the isolates. The most frequently identified bacteria were *Klebsiella pneumoniae*, *Escherichia coli*, *Pseudomonas aeruginosa*, and *Staphylococcus aureus*. Gram-negative bacilli were isolated in 85 (86%) cases, and resistance was present in 38 cases (44.7%), with both extended-spectrum beta-lactamase (ESBL) and *Klebsiella pneumoniae* Carbapenemase (KPC) detected in nine (10.5%). In 53 (34.4%) episodes, some complications occurred during FN. The 30-day all-cause mortality was 53 (34.4%), with 27 (50.9%) of these cases associated with complications. Of the 45 (86.5%) patients who died from any cause, all did so during their first episode of FN. In the bivariate analysis, the following factors were associated with higher mortality: hypertension (OR 2.58, 95% CI 1.19-5.58; p=0.014), chronic obstructive pulmonary disease (COPD) (OR 10.2, 95% CI 1.11-93.8; p=0.013), chronic kidney disease (OR 4.27, 95% CI 0.975-18.7; p=0.038), prolonged neutropenia (OR 2.34, 95% CI 1.1-4.95; p=0.024), and lactate dehydrogenase (LDH) levels greater than two times the upper normal limit (UNL) (OR 3.24, 95% CI 1.35-7.75; p=0.007). In contrast, normal albumin levels before chemotherapy were associated with lower mortality (OR 0.381, 95% CI 0.15-0.95; p=0.036). In the multivariate analysis, none of the identified factors were statistically significant in predicting complications or mortality.

Conclusion

No factors related to complications or mortality were found in the multivariate analysis. However, the heterogeneity of the population suggests that these outcomes are not determined by a single factor, and a study with a larger sample may be needed to confirm them.

## Introduction

Febrile neutropenia (FN) is defined by the presence of an absolute neutrophil count (PMN) below 500 cells/mm³ or less than 1000 cells/mm³ with a decrease expected lower than 500 cells/mm³ in the next 48 hours and body temperatures greater than 38.3°C in a single measurement, or 38°C sustained for at least one hour [1-4]. 

FN is a common and complex emergency in patients with hematological malignancies (HM) receiving chemotherapy, associated with several complications such as increased costs, prolonged hospitalizations, and mortality [1-3]. It is estimated that between 60% and 85% of patients will develop it at some point during treatment [1, 2, 5]. FN can be caused or perpetuated by bacterial, fungal, viral, and even parasitic infections, with bacterial infections being the most frequent [1, 5].

The management of these patients represents a great challenge for the health staff since the lack of adequate antimicrobial coverage against the germ can cause death while prolonged exposure to antibiotics leads to the selection of multiresistant microorganisms. In addition, FN can be caused by non-infectious factors [6, 7]. On the other hand, its coexistence with patient-specific factors or their underlying disease contributes to an increased risk of serious complications. In the 1960s and 1970s, most infections were caused by predominantly gram-negative bacilli (GNB), especially *Pseudomonas aeruginosa*, *Klebsiella pneumoniae*, and *Escherichia coli*, which were associated with high mortality rates, close to 50% [1, 3]. These findings led to the use of empiric antibiotic therapy, resulting in a drastic decrease in the mortality rate of neutropenic cancer patients [2-4].

A study by Garzón et al. [6] demonstrated a clear predominance of gram-negative bacteria, consistent with other studies conducted in Latin America [7, 8]. In the 1980s, a shift to a predominance of gram-positive cocci was observed [1, 5, 7], which continued into the 1990s with an increase in serious infections caused by the *Streptococcus viridans* group in neutropenic patients with acute leukemia undergoing hematopoietic stem cell transplantation or chemotherapy [1, 5, 9]. However, in recent years, infections caused by gram-negative bacteria have re-emerged [3, 7]. The epidemiological patterns of infections in neutropenic patients show periodic changes and are influenced by several factors, including the severity and duration of neutropenia, the nature and intensity of antineoplastic therapy, host-related characteristics, selective pressure from the use of prophylactic antibiotics or empirical antimicrobial therapy, the use of central catheters, environmental factors, and the duration of hospital stay [1, 3]. It is likely that these factors contribute to the development of antibiotic resistance mechanisms, which are important for defining the best empirical antimicrobial regimen for each institution. Some of these factors have been associated with an increased risk of mortality in these patients [7, 10]. There are therapeutic strategies based on risk stratification in these patients, which help estimate the probability of complications and mortality during an FN episode, such as the MASCC score [11], which, as noted, was validated in patients with not only hematological malignancies but also solid tumors.

Despite the high prevalence of HM in Colombia, there are few local studies that have evaluated the clinical and microbiological aspects of FN episodes [6, 12]. To our knowledge, there are no local analytical studies that have explored risk factors associated with mortality and complications in this context, highlighting the need for this study. For this reason, we decided to conduct this study with the aim of describing and characterizing FN episodes, as well as analyzing the presence of risk factors related to ICU admission, the need for vasopressors, mechanical ventilation, and death in these patients.

## Materials and methods

Study design

A retrospective cohort study was conducted based on a convenience sample of hospitalized patients at the Alma Mater Hospital of Antioquia (HAMA) and the San Vicente Foundation University Hospital (HUSVF), both referral centers for malignant hematological diseases. Patients were recruited between January 1st, 2018, and December 31st, 2020. The study was considered minimal risk research according to Colombian regulations and was approved by the research ethics committees of both institutions. Informed consent from the patients was not required due to the nature of the study.

Population

All episodes in patients over 18 years old with a diagnosis of HM and FN were included. FN was defined as a temperature greater than 38.3°C on one occasion or greater than 38°C sustained for 1 hour, with an absolute neutrophil count (ANC) of less than 500 cells/mm³ or less than 1000 cells/mm³ with a predicted decrease of less than 500 cells/mm³ in the following 48 hours at the time of diagnosis, or up to 30 days after receiving chemotherapy as part of the treatment for the underlying HM. Patients were excluded if the primary outcome could not be assessed due to incomplete information (e.g., no record of the hospitalization site, use of mechanical ventilation or vasopressors) or referral to another institution before outcomes such as mortality could be assessed. Additionally, those who had a bacterial infection within 30 days prior to admission, caused by a clinical condition unrelated to FN, were excluded.

Variables

Clinical, paraclinical, and microbiological variables were determined. Clinical variables included age, comorbidities, type of HM, type of chemotherapy, use of granulocyte colony-stimulating factors, functional status, MASCC score, and days with severe neutropenia. The MASCC score was calculated retrospectively, and inter-rater reliability was addressed by rechecking the data with different data collectors. Paraclinical variables included factors such as C-reactive protein (CRP), ferritin, coagulation times, lactate dehydrogenase (LDH), and creatinine. Regarding microbiological variables, clinical infection sites, isolated microorganisms, antimicrobial agents used, and the presence or absence of antimicrobial prophylaxis were recorded.

Outcomes

The primary outcomes were defined as severe complications and mortality within the first 30 days of FN. Severe complications were defined as ICU admission, requirement for mechanical ventilation, and need for vasopressor support. Mortality was evaluated both during hospitalization for any cause and during episodes of severe neutropenia.

Source

The information was collected through a review of the electronic medical records of patients with HM according to the CIE-10 diagnostic code. All episodes of FN in each patient were considered. Laboratory parameters were collected at the time of the FN episode.

Bias control

Information biases were mitigated by clearly defining each variable to be evaluated for the patients, and the support staff responsible for data collection was trained prior to the study's implementation. A double-checking process was carried out by the principal investigator to ensure accuracy. Missing data was handled by removing with deleting rows and columns. 

Statistical analysis

In the data analysis, for the descriptive component, measures of central tendency such as the mean with its standard deviation or the median with its interquartile range were used for quantitative variables, depending on the data distribution determined by the Shapiro-Wilk test. For qualitative variables, absolute and relative frequency distributions of the variable categories were used.

For the analytical component, the outcomes were defined as having any complication and dying from any cause. A bivariate analysis was performed, relating the independent variables to both outcomes by calculating the chi-square (X²) test and p-value for discrete variables, and the Student’s t-test and p-value for quantitative variables. We chose p ≤ 0.25 for inclusion in the multivariate analysis because we wanted to retain potentially informative variables while avoiding overly strict criteria that might discard valuable predictors. If the differences were statistically significant, the odds ratio (OR) and its confidence interval were calculated. Multivariate analysis was conducted using binary logistic regression, including variables with a p-value ≤ 0.25. For the data analysis, the statistical software packages JASP and JAMOVI were used.

## Results

Epidemiological and clinical characteristics

**Figure 1:**
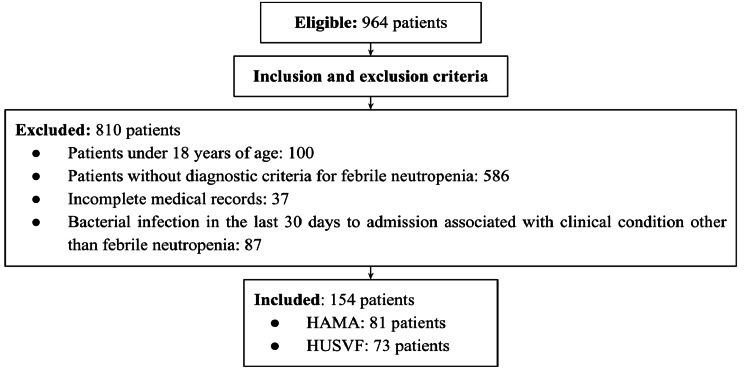
Patient recruitment flowchart HAMA, Alma Mater Hospital of Antioquia; HUSVF, San Vicente Foundation University Hospital

Out of 964 eligible patients, 154 (15.97%) were selected after applying the exclusion criteria (Figure 1), and a total of 190 episodes of FN were recorded.

The median age was 53 years. Of the total patients, 83 (53.9%) were male. The most frequently found HM were acute myeloid leukemia, acute lymphoid leukemia, and large B-cell lymphoma, representing 52 (33.8%), 42 (27.3%), and 27 (17.5%) of the patients, respectively. The most common comorbidities were hypertension in 37 patients (24.02%), diabetes mellitus in 17 patients (11.04%), and dyslipidemia in 16 patients (10.4%). Patients with an ECOG score >2 represented 47 patients (24.7%), and 98 patients (51.6%) had a MASCC score <21. At the time of the diagnosis of FN, the median hospital stay was 20 days, and the median duration of severe neutropenia, when it occurred, was 10 days. The sociodemographic data and underlying diseases of the patients are shown in Table 1.

**Table 1:** Clinical characteristics of patients with hematologic malignancy and FN ♦Normal distribution assessed by the Shapiro-Wilk test: data are presented as mean and standard deviation. Other data are presented as median and interquartile range. ^§^More antineoplastic therapies are observed than patients because the chemotherapy regimen could change in the same patient from one episode to another. ^¶¶^The number does not correlate with the total number of FN episodes because each patient could receive more than one drug. Each drug was related to the total number of FN episodes. ♣Data obtained from the total number of FN episodes (n=190). FN, febrile neutropenia; COPD, chronic obstructive pulmonary disease; HCO_3_-, bicarbonate; PaO_2_/FiO_2_, ratio of the partial pressure of arterial oxygen to the fraction of inspired oxygen; CRP, C-reactive protein; PT, prothrombin time; PTT, partial thromboplastin time; LDH, lactate dehydrogenase

Characteristics	Patients (n=154), n (%)
Age in years ^♣^, median (range)	53 (18-91)
Sex, n (%)	
Male	83 (53.9)
Female	71 (46.1)
Comorbidities, n (%)	
Arterial hypertension	37 (24.02)
Diabetes mellitus	17 (11.04)
Dyslipidemia	16 (10.4)
Immunosuppression	12 (7.8)
Autoimmune disease	8 (5.2)
Chronic kidney disease	8 (5.2)
Obesity	6 (3.9)
Active solid organ malignancy	6 (3.9)
COPD	5 (3.2)
Cirrhosis	2 (1.3)
Hematologic malignancy, n (%)	
Acute myeloid leukemia	52 (33.8)
Acute lymphocytic leukemia	42 (27.3)
Diffuse large B-cell lymphoma	27 (17.5)
Burkitt lymphoma	9 (5.8)
Mantle cell lymphoma	5 (3.2)
Multiple myeloma	4 (2.6)
Non-Hodgkin lymphoma	4 (2.6)
Lymphoblastic lymphoma	3 (1.9)
Hodgkin lymphoma	3 (1.9)
T-cell lymphoma	2 (1.3)
Chronic lymphocytic leukemia	1 (0.6)
Myelofibrosis	1 (0.6)
Chronic myeloid leukemia in therapy	1 (0.6)
Type of chemotherapy^§,♣^, n (%)	
Acute leukemias	132 (69.5)
Lymphoproliferative neoplasms	48 (25.3)
Myeloproliferative neoplasms	2 (1.05)
Plasma cell neoplasms	2 (1.05)
Allogeneic/autologous transplantation	6 (3.2)
Use of granulocyte-macrophage colony-stimulating factor^♣^, n (%)	118 (62.105)
Charlson comorbidity index^♣^, median (range)	2 (0-26)
ECOG performance status ≥2^♣^, n (%)	47 (24.7)
MASCC <21 points^♣^, median (%)	98 (51.6)
Days with neutropenia since diagnosis of severe neutropenia (<500 cells/mm^3^)^♣^, median (range)	10 (0-68)
Days on antibiotics since diagnosis of FN^♣^, median (range)	13 (0-70)
Days of hospital stay since FN was documented^♣^, median (range)	20 (0-134)
Clinical characteristics at FN day diagnosis^♣^, median (range)	
Hemoglobin, g/dL (range)	8.25 (4.1-17.5)
Neutrophil absolute count, cells/mm^3^ (range)	70 (0-3300)
Platelets, plt/mm^3^ (range)	25000 (357-265000)
Arterial pH, SCALE (range)	7.4 (7.18-7.62)
HCO_3_-^♦^, mEq/L (range)	21.79 (±4.32)
PaO_2_/FiO_^2^_, mmHg/% (range)	198.6 (83-610)
CRP, mg/dL (range)	9.3 (0.020-49.07)
Ferritin, ng/mL (range)	2076.4 (81.16-57564)
PT, seg (range)	12.5 (10-24.2)
PTT, seg (range)	32.9 (20.6-77.2)
Fibrinogen, mg/dL (range)	446.6 (142-1060)
LDH, U/L (range)	222.6 (1.39-4145)
Creatinine, mg/dL (range)	0.71 (0.200-2.07)
Seric albumin^♦^, g/dL (range)	3.02 (±0.614)
Empiric antibiotic therapy^¶¶^, n (%)	
Cefepime	94 (49.5)
Piperacillin/tazobactam	66 (34.7)
Fluconazole	62 (32.6)
Vancomycin	57 (30)
Meropenem	31 (16.4)
Trimethoprim-sulfamethoxazole	10 (5.3)
Amikacin	9 (4.7)
Antibiotic prophylaxis^♣^, n (%)	
Antiviral	134 (70.5)
Antifungal	129 (67.9)

Frequency of infections and pathogen distribution

**Figure 2:**
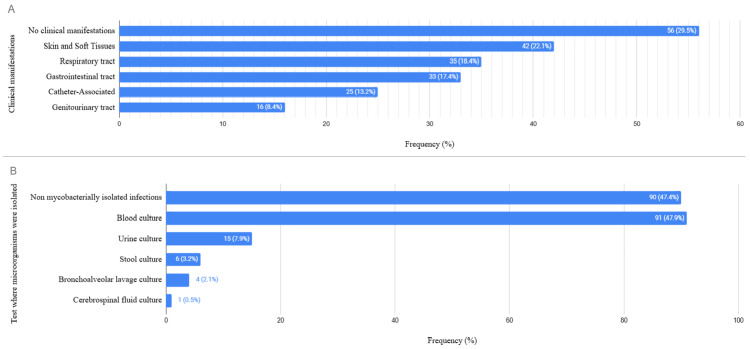
Distribution of infection and isolation sites A. Clinical manifestations suggestive of infection identified in patients at the time of FN episode diagnosis. B. Tests performed on patients in whom at least one microorganism was identified. The percentages represented were obtained from the total number of FN episodes (n=190). FN, febrile neutropenia

FN occurred during or after chemotherapy exposure in 164 (86.3%) cases. Of the total episodes of FN, 118 (62.1%) patients had received granulocyte colony-stimulating factors prior to the onset of the oncological emergency. In 134 (70.5%) of the FN episodes, an infectious focus was clinically documented. Infections were reported in soft tissues in 42 (22.1%) episodes, the respiratory system in 35 (18.4%), the gastrointestinal system in 33 (17.4%), associated with catheters in 25 (13.2%), and in the genitourinary tract in 16 (8.4%) (Figure 2A). A causative agent was identified in 125 episodes (65.8%), with the majority being bacteria in 112 cases (92.6%) of the isolates (Table 2). The most frequent microorganisms were Klebsiella pneumoniae in 31 cases (24.8%), *Escherichia coli* in 27 cases (21.6%), *Pseudomonas aeruginosa* in 12 cases (9.6%), and *Staphylococcus aureus* in 12 cases (9.6%) (Table 2). The frequency of antimicrobial resistance in GNB was 38 cases (44.7%), and resistance due to mobile genetic elements was found in nine cases (10.5%) for both ESBL and KPC. In gram-positive cocci, seven (26.7%) were resistant to oxacillin. These pathogens were primarily isolated in blood cultures in 91 cases (47.9%), urine cultures in 15 patients (7.9%), and stool cultures in six cases (3.2%) (Figure 2B).

**Table 2:** Frequency of isolated microorganisms **Catheter-related. ^○^They were bacteria isolated from patients who died before microbiological identification. FN, febrile neutropenia

Isolated microorganism	Infection cases (n=125), n (%)
Bacteria, n (%)	112 (92.6)
Gram-negative, n (%)	85 (86)
Klebsiella pneumoniae	31 (24.8)
Escherichia coli	27 (21.6)
Pseudomonas aeruginosa	12 (9.6)
Serratia marcescens	3 (2.4)
*Acinetobacter Baumannii *complex	3 (2.4)
Others^○^	9 (7.2)
Gram-positive, n (%)	27 (21.6)
*Staphylococcus aureus* (4**)	12 (9.6)
*Staphylococcus epidermidis* (3**)	3 (2.4)
Clostridium difficile	2 (1.6)
Others^○^	10 (8)
Fungi, n (%)	7 (5.78)
*Candida parapsilosis* (1**)	2 (1.6)
Candida tropicalis	3 (2.4)
Candida albicans	1 (0.8)
Rhodotorula mucilaginosa	1 (0.8)**
Virus, n (%)	2 (1.65)
Herpes Simplex virus	1 (0.8)
Epstein-Barr virus	1 (0.8)
Others^○^, n (%)	4 (3.2)

The most used empirical antimicrobial therapy was cefepime in 94 cases (49.5%) and piperacillin/tazobactam in 66 cases (34.7%), with limited use of empirical antifungal therapy. Antiviral and antifungal prophylaxis were present in 134 (70.5%) and in 129 (67.9%) of cases, respectively. No patient received antibacterial prophylaxis.

Outcomes

Regarding the clinical outcomes of interest, 53 patients (34.4%) experienced some complications (Table 3). In terms of mortality, intrahospital death from any cause was reported in 53 patients (34.4%). Of these, 27 patients (50.9%) who had complications related to FN were among those who died. Additionally, 41 patients (26.6%) died while in severe neutropenia (ANC <500 cells/mm³) (Table 3). Among those who died from any cause, 45 patients (86.5%) did so during their first episode of FN (Table 4 and Table 5).

**Table 3:** Clinical outcomes assessed within episodes of FN per patient FN, febrile neutropenia

Outcome	Frequency (n=154), n (%)
Admission to intensive care unit and/or requirement for vasopressor support and/or requirement for mechanical ventilation	53 (34.4)
In-hospital mortality from all causes	53 (34.4)
Mortality during neutropenia	41 (26.6)

**Table 4:** Number of episodes of FN in patients who died FN, febrile neutropenia

Total episodes	In-hospital mortality from all causes (n=53), n (%)	Mortality during severe neutropenia (n=41), n (%)
1^st ^episode	45 (86.5)	36 (87.8)
2^nd^ episode	4 (7.7)	3 (7.3)
3^rd ^episode	2 (3.8)	1 (2.4)
4^th ^episode	1 (1.9)	1 (2.4)

**Table 5:** Complicated and/or deceased vs. uncomplicated and alive ^♦^Normal distribution assessed by the Shapiro-Wilk test: data are presented as mean and standard deviation. Other data are presented as median and interquartile range. ^§^More antineoplastic therapies are observed than the number of patients because the chemotherapy regimen may change for the same patient from one episode to another. ^¶¶^The number does not correlate with the total number of FN episodes because each patient may receive more than one drug. Each drug is associated with the total number of FN episodes. ^♣^Data obtained from the total number of FN episodes (n=190). FN, febrile neutropenia; COPD, chronic obstructive pulmonary disease; HCO_3_-, bicarbonate; PaO_2_/FiO_2_, ratio of the partial pressure of arterial oxygen to the fraction of inspired oxygen; CRP, C-reactive protein; PT, prothrombin time; PTT, partial thromboplastin time; LDH, lactate dehydrogenase

Characteristics	Complicated and/or death (n=76), n (%)	Uncomplicated and alive (n=78), n (%)
Age in years^♣^, median (range)	56.5 (18-91)	48 (18-83)
Sex, n (%)		
Male	46 (60.5)	37 (47.4)
Female	30 (39.5)	41 (52.6)
Comorbidities, n (%)		
Arterial hypertension	20 (27.8)	17 (20.7)
Diabetes mellitus	8 (11.1)	9 (11.0)
Dyslipidemia	9 (12.5)	7 (8.5)
Immunosuppression	7 (9.7)	5 (6.1)
Autoimmune disease	5 (6.9)	3 (3.7)
Chronic kidney disease	6 (8.3)	2 (2.4)
Obesity	3 (4.2)	3 (3.7)
Active solid organ malignancy	4 (5.6)	2 (2.4)
COPD	5 (6.9)	0 (0)
Cirrhosis	1 (1.4)	1 (1.2)
Hematologic malignancy, n (%)		
Acute myeloid leukemia	24 (31.6)	28 (35.89)
Acute lymphocytic leukemia	16 (21.05)	26 (33.33)
Diffuse large B-cell lymphoma	10 (13.2)	17 (21.8)
Burkitt lymphoma	2 (2.63)	7 (8.97)
Mantle cell lymphoma	3 (3.95)	2 (2.6)
Multiple myeloma	2 (2.63)	2 (2.6)
Non-Hodgkin lymphoma	3 (3.95)	1 (1.3)
Lymphoblastic lymphoma	1 (1.32)	2 (2.6)
Hodgkin lymphoma	1 (1.32)	2 (2.6)
T-cell lymphoma	1 (1.32)	1 (1.3)
Chronic lymphocytic leukemia	1 (1.32)	0 (0)
Myelofibrosis	0 (0)	1 (1.3)
Chronic myeloid leukemia in therapy	1 (1.32)	0 (0)
Type of chemotherapy^§,♣^, n (%)		
Acute leukemias	62 (81.6)	70 (89.7)
Lymphoproliferative neoplasms	14 (18.42)	34 (43.6)
Myeloproliferative neoplasms	2 (2.63)	0 (0)
Plasma cell neoplasms	2 (2.63)	0 (0)
Allogeneic/autologous transplantation	2 (2.63)	4 (5.13)
Use of granulocyte-macrophage colony-stimulating factor^♣^, n (%)	46 (56.1)	72 (66.7)
Charlson comorbidity index^♣^, median (range)	3 (1-26)	2 (0-26)
ECOG performance status ≥2^♣^, n (%)	27 (35.5)	20 (25.6)
MASCC < 21 points^♣^, median (%)	51 (62.2)	47 (43.5)
Days with neutropenia since diagnosis of severe neutropenia (<500 cells/mm^3^)^♣^, median (range)	14 (0-68)	8 (1-61)
Days on antibiotics since diagnosis of FN^♣^, median (range)	15 (0-70)	11 (2-54)
Days of hospital stay since FN was documented^♣^, median (range)	20.5 (0-99)	19.5 (3-134)
Clinical characteristics at FN day diagnosis^♣^, median (range)		
Hemoglobin, g/dL (range)	7.9 (4.1-12.6)	8.35 (5.7-17.5)
Neutrophil absolute count, cells/mm^3^ (range)	85 (0-3300)	45 (0-500)
Platelets, plt/mm^3^ (range)	22500 (357-265000)	28000 (1500-246000)
Arterial pH, SCALE (range)	7.45 (7.18-7.6)	7.47 (7.4-7.62)
HCO_3_-^♦^, mEq/L (range)	21.44 (±5.08)	22.34 (±2.85)
PaO_2_/FiO_2_, mmHg/% (range)	245 (83-500)	182.29 (144-610)
CRP, mg/dL (range)	8.92 (0.11-49.07)	9.28 (0.02-37.1)
Ferritin, ng/mL (range)	2936 (581.4)	1620.3 (81.16-3520.3)
PT, seg (range)	12.5 (10.3-24.2)	12.4 (10-19)
PTT, seg (range)	35.8 (20.6-77.2)	31 (21-45)
Fibrinogen, mg/dL (range)	449.38 (142-944)	399.15 (210.3-1060)
LDH, U/L (range)	285.8 (1.39-1897)	164 (94-4145)
Creatinine, mg/dL (range)	0.83 (0.2-13.4)	0.640 (0.33-20.7)
Seric albumin^♦^, g/dL (range)	2.87 (1.8-4.01)	3.18 (±0.66)
Empiric antibiotic therapy^¶¶^, n (%)		
Cefepime	33 (40.2)	61 (56.5)
Piperacillin/tazobactam	33 (40.2)	33 (30.6)
Fluconazole	34 (41.5)	28 (25.9)
Vancomycin	31 (37.8)	26 (24.1)
Meropenem	9 (11.0)	22 (20.4)
Trimethoprim-sulfamethoxazole	7 (9.21)	3 (3.85)
Amikacin	7 (9.21)	2 (2.6)
Antibiotic prophylaxis^♣^, n (%)		
Antiviral	62 (75.6)	72 (66.7)
Antifungal	60 (73.2)	69 (63.9)
In-hospital mortality from all causes, n (%)	53 (69.7)	0 (0)

Factors related to complications

Patients were divided into those with severe complications (ICU admission, need for vasopressors, or need for mechanical ventilation) and those without complications. The sociodemographic characteristics evaluated in these groups are described in Table 6. In the bivariate analysis, elevated LDH levels greater than two times the upper normal limit (UNL) at the time of the FN diagnosis were associated with an increased risk of complications, while a MASCC score >21 was associated with a lower probability of complications (Table 7). Neither of these two factors was statistically significant in the multivariate analysis.

**Table 6:** Clinical characteristics of patients with HM and FN: complicated vs. uncomplicated ♦Normal distribution assessed by the Shapiro-Wilk test: data are presented as mean and standard deviation. Other data are presented as median and interquartile range. §More antineoplastic therapies are observed than the number of patients because the chemotherapy regimen may change for the same patient from one episode to another. ¶¶The number does not correlate with the total number of FN episodes because each patient may receive more than one drug. Each drug is associated with the total number of FN episodes. ♣Data obtained from the total number of FN episodes (n=190). FN, febrile neutropenia; COPD, chronic obstructive pulmonary disease; HCO3-, bicarbonate; PaO2/FiO2, ratio of the partial pressure of arterial oxygen to the fraction of inspired oxygen; CRP, C-reactive protein; PT, prothrombin time; PTT, partial thromboplastin time; LDH, lactate dehydrogenase; HM, hematological malignancy

Characteristics	Complicated: admission to ICU and/or vasopressor requirement and/or mechanical ventilation requirement (n=53), n (%)	Uncomplicated (n=101), n (%)
Age in years^♣^, median (range)	54 (18-79)	52 (18-91)
Sex, n (%)		
Male	30 (60)	53 (51)
Female	20 (40)	51 (49)
Comorbidities, n (%)		
Arterial hypertension	12 (24)	25 (24)
Diabetes mellitus	5 (10)	12 (11.5)
Dyslipidemia	4 (8)	12 (11.5)
Immunosuppression	6 (12)	6 (5.8)
Autoimmune disease	3 (6)	5 (4.8)
Chronic kidney disease	3 (6)	5 (4.8)
Obesity	3 (6)	3 (2.9)
Active solid organ malignancy	3 (6)	3 (2.9)
COPD	2 (4)	3 (2.9)
Cirrhosis	1 (2)	1 (1)
Hematologic malignancy, n (%)		
Acute myeloid leukaemia	16 (32)	36 (34.6)
Acute lymphocytic leukemia	15 (30)	27 (26)
Diffuse large B-cell lymphoma	6 (12)	21 (20.2)
Burkitt lymphoma	3 (6)	6 (5.8)
Mantle cell lymphoma	2 (4)	3 (2.9)
Multiple myeloma	2 (4)	2 (1.9)
Non-Hodgkin lymphoma	2 (4)	2 (1.9)
Lymphoblastic lymphoma	1 (2)	2 (1.9)
Hodgkin lymphoma	1 (2)	2 (1.9)
T-cell lymphoma	1 (2)	1 (0.96)
Chronic lymphocytic leukemia	1 (2)	0 (0)
Myelofibrosis	0 (0)	1 (0.96)
Chronic myeloid leukemia in therapy	0 (0)	1 (0.96)
Type of chemotherapy^§,♣^, n (%)		
Acute leukemias	37 (66.07)	95 (70.9)
Lymphoproliferative neoplasms	16 (28.6)	32 (23.9)
Myeloproliferative neoplasms	0 (0)	2 (1.5)
Plasma cell neoplasms	2 (3.6)	0 (0)
Allogeneic/autologous transplantation	1 (1.8)	5 (3.7)
Use of granulocyte-macrophage colony-stimulating factor^♣^, n (%)	32 (57.1)	86 (64.2)
Charlson comorbidity index^♣^, median (range)	3 (1-26)	2 (0-26)
ECOG performance status ≥2^♣^, n (%)	20 (35.7)	27 (20.1)
MASCC < 21 points^♣^, median (%)	37 (66.1)	61 (45.5)
Days with neutropenia since diagnosis of severe neutropenia (<500 cells/mm^3^)^♣^, median (range)	10 (0-46)	10.5 (0-68)
Days on antibiotics since diagnosis of FN (median (range)^♣^, n (range)	14.5 (0-70)	11 (0-60)
Days of hospital stay since FN was documented (median (range)^♣^, n (range)	21.5 (0-99)	19 (0-134)
Clinical characteristics at FN day diagnosis^♣^, median (range)		
Hemoglobin, g/dL (range)	7.95 (5-12.6)	8.3 (4.1-17.5)
Neutrophil absolute count, cells/mm^3^ (range)	80 (0-3300)	55 (0-600)
Platelets, plt/mm^3^ (range)	23000 (357-260000)	27000 (1500-265000)
Arterial pH, SCALE (range)	7.45 (7.18-7.60)	7.47 (7.4-7.62)
HCO_3_-^♦^, mEq/L (range)	21.13 (±5.05)	22.70 (±3.02)
PaO_2_/FiO_2_, mmHg/% (range)	212.4 (83-500)	198.57 (144-610)
CRP, mg/dL (range)	8.9 (0.110-49.07)	9.28 (0.02-37.10)
Ferritin, ng/mL (range)	2427.2 (1152-12454.7)	1971.4 (81.16-57564)
PT, seg (range)	12.5 (10.3-24.2)	12.5 (10-19.5)
PTT, seg (range)	35.55 (20.6-77.2)	32.6 (21-46.5)
Fibrinogen, mg/dL (range)	529.67 (142-944)	443.76 (210.3-1060)
LDH, U/L (range)	256.85 (1.39-1897)	183.05 (94-4145)
Creatinine, mg/dL (range)	0.8 (0.200-13.4)	0.67 (0.32-20.7)
Seric albumin^♦^, g/dL (range)	2.87 (±0.57)	3.12 (±0.63)
Empiric antibiotic therapy^¶¶^, n (%)		
Cefepime	22 (39.3)	72 (53.7)
Piperacillin/tazobactam	22 (39.3)	44 (32.8)
Fluconazole	22 (39.3)	40 (29.9)
Vancomycin	17 (30.4)	40 (29.8)
Meropenem	5 (8.9)	26 (19.4)
Trimethoprim-sulfamethoxazole	8 (14.3)	2 (1.5)
Amikacin	1 (1.8)	8 (6)
Antibiotic prophylaxis^♣^, n (%)		
Antiviral	41 (73.2)	93 (69.4)
Antifungal	39 (69.6)	90 (67.2)
In-hospital mortality from all causes, n (%)	27 (54)	26 (25)

**Table 7:** Statistical analysis of qualitative variables associated with complications FN, febrile neutropenia; LDH, lactate dehydrogenase

Variable	Univariable analysis	p-value
MASCC	OR 0.46 IC 95% 0.24-0.861	0.014
Normal albumin post-chemotherapy	OR 0.09 IC 95% 0.004-1.9	0.042
High LDH prechemotherapy	OR 2.47 IC 95% 1.04-5.9	0.038
High LDH on FN day	OR 2.78 IC 95% 1.16-6.65	0.020

Factors related to mortality

The sociodemographic characteristics of those who died from any cause and those who survived are described in Table 8. In the bivariate analysis, the following factors were associated with higher mortality: hypertension (p=0.014), COPD (p=0.013), chronic kidney disease (p=0.038), prolonged neutropenia (p=0.024), LDH levels greater than two times the UNL (p=0.007), and normal albumin levels before chemotherapy (p=0.036). In contrast, higher albumin levels before chemotherapy were associated with lower mortality (Table 9). However, in the multivariate analysis, none of the identified factors were statistically significant.

**Table 8:** Multivariate analysis of qualitative variables related to all-cause mortality COPD, chronic obstructive pulmonary disease; LDH, lactate dehydrogenase; FN, febrile neutropenia; ICU, intensive care unit

Variable	Análisis univariable	p-value
Arterial hypertension	OR 2.58 IC 95% 1.19-5.58	0.014
COPD	OR 10.2 IC 95% 1.11-93.8	0.013
Chronic kidney disease	OR 4.27 IC 95% 0.975-18.7	0.013
Immunosuppression	OR 0.196 IC 95% 0.02-1.56	0.024
MASCC	OR 0.401 IC 95% 0.2-0.77	0.006
Prolonged neutropenia	OR 2.34 IC 95% 1.1-4.95	0.024
High LDH on FN day	OR 3.24 IC 95% 1.35-7.75	0.007
Normal albumin prechemotherapy	OR 0.381 IC 95% 0.15-0.95	0.036
Admission to ICU	OR 2.43 IC 95% 1.16-5.08	0.016
Vasopressor requirement	OR 6.2 IC 95% 2.94-13.0	<0.001
Mechanical ventilation requirement	OR 29.8 IC 95% 6.58-135	<0.001

**Table 9:** Sociodemographic characteristics of patients with hematologic malignancy and FN: death vs. survivors ♦Normal distribution assessed by the Shapiro-Wilk test: data are presented as mean and standard deviation. Other data are presented as median and interquartile range. §More antineoplastic therapies are observed than the number of patients because the chemotherapy regimen may change for the same patient from one episode to another. ¶¶The number does not correlate with the total number of FN episodes because each patient may receive more than one drug. Each drug is associated with the total number of FN episodes. ♣Data obtained from the total number of FN episodes (n=190). FN, febrile neutropenia; COPD, chronic obstructive pulmonary disease; HCO_3_-, bicarbonate; PaO_2_/FiO_2_, ratio of the partial pressure of arterial oxygen to the fraction of inspired oxygen; CRP, C-reactive protein; PT, prothrombin time; PTT, partial thromboplastin time; LDH, lactate dehydrogenase

Characteristics	Alive (n=102), n (%)	Death (n=53), n (%)
Age in years ^♣^, median (range)	47 (18-83)	58 (18-91)
Sex, n (%)		
Male	51 (50)	32 (61.5)
Female	50 (49)	21 (40.4)
Comorbidities, n (%)		
Arterial hypertension	20 (18.5)	17 (37.0)
Diabetes mellitus	11 (10.2)	6 (13.0)
Dyslipidemia	9 (8.3)	7 (15.2)
Immunosuppression	11 (10.2)	1 (2.2)
Autoimmune disease	4 (3.7)	4 (8.7)
Chronic kidney disease	3 (2.8)	5 (10.9)
Obesity	5 (4.6)	1 (2.2)
Active solid organ malignancy	4 (3.7)	2 (4.3)
COPD	1 (0.9)	4 (8.7)
Cirrhosis	2 (1.9)	0 (0.0)
Hematologic malignancy, n (%)		
Acute myeloid leukemia	32 (31.7)	20 (37.7)
Acute lymphocytic leukemia	36 (35.6)	6 (11.3)
Diffuse large B-cell lymphoma	23 (22.8)	4 (7.5)
Burkitt lymphoma	8 (7.9)	1 (1.9)
Mantle cell lymphoma	4 (3.96)	1 (1.9)
Multiple myeloma	2 (1.98)	2 (3.8)
Non-Hodgkin lymphoma	3 (2.97)	1 (1.9)
Lymphoblastic lymphoma	2 (1.98)	1 (1.9)
Hodgkin lymphoma	3 (2.97)	0 (0)
T-cell lymphoma	1 (0.99)	1 (1.9)
Chronic lymphocytic leukemia	0 (0)	1 (1.9)
Myelofibrosis	1 (0.99)	0 (0)
Chronic myeloid leukemia in therapy	0 (0)	1 (1.9)
Type of chemotherapy^§,♣^, n (%)		
Acute leukemias	94 (93.07)	38 (71.7)
Lymphoproliferative neoplasms	38 (37.6)	10 (18.9)
Myeloproliferative neoplasms	1 (0.99)	1 (1.9)
Plasma cell neoplasms	0 (0)	2 (3.8)
Allogeneic/autologous transplantation	4 (3.96)	2 (3.8)
Use of granulocyte-macrophage colony-stimulating factor^♣^, n (%)	89 (65.0)	29 (54.7)
Charlson comorbidity index^♣^, median (range)	2 (0-26)	3 (1-26)
ECOG performance status ≥2^♣^, n (%)	27 (26.7)	20 (37.7)
MASCC <21 points^♣^, median (%)	63 (46.0)	35 (66.0)
Days with neutropenia since diagnosis of severe neutropenia (<500 cells/mm^3^)^♣^, median (range)	8 (1-61)	15.5 (0-68)
Days on antibiotics since diagnosis of FN^♣^, median (range)	12 (2-54)	14 (0-70)
Days of hospital stay since FN was documented^♣^, median (range)	23 (3-134)	16 (0-99)
Positive bloodstream cultures, n (%)	45 (44.11)	31 (59.61)
Clinical characteristics at FN day diagnosis^♣^, median (range)		
Hemoglobin, g/dL (range)	8.3 (5.7-17.5)	8.1 (4.1-10)
Neutrophil absolute count, cells/mm^3^ (range)	40 (0-3300)	100 (0-600)
Platelets, plt/mm^3^ (range)	26000 (357-246000)	18000 (3000-265000)
Arterial pH, SCALE (range)	7.46 (7.4-7.62)	7.46 (7.18-7.54)
HCO_3_-^♦^, mEq/L (range)	22.05 (±3.15)	21.33 (±6.07)
PaO_2_/FiO_^2^_, mmHg/% (range)	245 (129.5-610)	175.53 (83-439.04)
CRP, mg/dL (range)	7.88 (0.02-49.07)	11.56 (0.11-34.7)
Ferritin, ng/mL (range)	1868 (81.16-3655)	3377 (581.4-57564)
PT, seg (range)	12.25 (10-24.2)	12.7 (10.7-19.6)
PTT, seg (range)	32.2 (21-77.2)	35.05 (20.6-50.7)
Fibrinogen, mg/dL (range)	414 (142-1060)	449.38 (228-944)
LDH, U/L (range)	174 (94-4145)	287.3 (1.39-1897)
Creatinine, mg/dL (range)	0.69 (0.2-20.7)	0.87 (0.32-13.4)
Seric albumin^♦^, g/dL (range)	3.15 (±0.6)	2.85 (±0.61)
Empiric antibiotic therapy^¶¶^, n (%)		
Cefepime	74 (54.0)	20 (37.7)
Piperacillin/tazobactam	44 (32.1)	22 (41.5)
Fluconazole	39 (28.5)	23 (43.4)
Vancomycin	34 (24.8)	23 (43.4)
Meropenem	24 (17.5)	7 (13.2)
Trimethoprim-sulfamethoxazole	3 (2.97)	7 (13.2)
Amikacin	3 (2.97)	6 (11.3)
Antibiotic prophylaxis^♣^, n (%)		
Antiviral	94 (68.6)	40 (75.5)
Antifungal	91 (66.4)	38 (71.7)

## Discussion

To our knowledge, this is the first study conducted at two referral centers in Colombia that evaluate clinical factors related to complications and mortality in patients with HM and FN within the first 30 days after diagnosis and treatment initiation, providing valuable information for medical decision-making. Patients undergoing treatment for HM are at imminent risk of life-threatening infections due to immune system dysfunction associated with the disease and chemotherapy-induced neutropenia [13]. It is well-established that FN is an oncological emergency with high mortality that requires early antimicrobial intervention [1-3].

After the shift from gram-negative to gram-positive pathogens observed worldwide during the 1980s, gram-positive bacteria became predominant in most developed countries, while developing countries still show a predominance of gram-negative bacteria, possibly due to less antibiotic prophylaxis [14]. However, in our study of non-transplanted patients undergoing NH treatment, most infectious complications were bacterial in 112 (89.6%) episodes, with pathogens isolated from blood cultures in 56 (50%) cases, and gram-negative bacteria causing 85 (75.89%) of those cases. The microbiological confirmation and proportion of gram-negative bacteria were higher than in previously published data, where it ranged from 40% to 50% [14, 15]. This can be explained by intestinal mucosal injury caused by chemotherapy, which leads to bacteremia from gram-negative bacteria originating from the intestinal flora. During the years covered in our study, antibiotic prophylaxis was not typically administered [3]. Historically, there has been a high risk of infection from gram-positive cocci. A previous study found that gram-positive bacteria represented 26% (n=203) of cases in acute leukemia patients with FN [15], a figure close to our study, which identified 27 isolates (21.6%), as shown in Table 2. *Staphylococcus aureus* was the most frequent gram-positive coccus, with 12 (9.6%) isolates. Therefore, these findings support the decision to initiate empirical antibiotic treatment directed against gram-negative microorganisms, including *Pseudomonas aeruginosa*, and to use anti-gram-positive cocci antimicrobials only in high-risk scenarios for infections by these microorganisms. Fungal infections were documented in only seven (5.78%) cases, similar to other studies [16], although this was lower in frequency than in studies where active searches for such infections were performed [17]. The frequency of positive blood cultures, which in other studies has been reported between 11% and 38% [18], was 91 (47.9%) in our study. This could be explained by a higher infection rate associated with catheters, which had a frequency of 25 (13.2%) in our study, very similar to the 9% (n=33) found by Sereeaphinan et al. (2021) [19], where primary bacteremia was 40% (n=33). However, it should be acknowledged that historically, it has been difficult to distinguish catheter-related bacteremias from those originating in the intestine, an entity now known as mucosal barrier injury associated with confirmed bacteremia [3]. In 125 (65.8%) episodes, a pathogen was isolated, which is higher than in another study where the proportion was 34% (n=827) [8].

Patients with NH who were exposed to chemotherapy and had a worse functional status according to the ECOG scale generally experienced higher complication and mortality rates [8, 20-22], as was the case in our study. Regarding laboratory parameters, contrary to some studies, we did not find significant differences in the frequency of anemia between those who developed severe complications and those who did not [20]. However, hypoalbuminemia was more common in patients with FN who had complications [23]. As for the use of antimicrobial prophylaxis, there was no difference between the two groups. However, the literature suggests that prophylaxis not only reduces infection rates and subsequent FN episodes but also severe complications, as reported by Demirel et al. (2015) and Cullen et al. (2009) over a decade ago [24, 25]. In the bivariate analysis, elevated LDH levels at the time of FN were associated with severe complications, a finding already reported by Karimi et al. (2018) [26]. Additionally, hypoalbuminemia was identified as a risk factor, as some reports have found that albumin levels <3.5 g/dL increase the incidence of FN (Table 7) [27].

In different studies, afebrile neutropenia had a mortality rate between 6% and 35% of cases [15, 27], while in our study, mortality from any cause was 53 (34.4%), with 41 (77.3%) cases occurring during FN, and 36 (67.9%) deaths from any cause and during severe neutropenia occurring during the first episode. This data has not been directly described in the literature. However, it has been reported, primarily in solid neoplasms, that about 60% of FN episodes occur during the first chemotherapy cycle [3, 28]. The mortality rate was higher than that reported in other studies with similar populations. One factor we believe plays a role is the high prevalence of antimicrobial resistance, with nine cases (10.5%) for both KPC and ESBL, and the fact that both centers observed inappropriate empirical antimicrobial coverage for the resistance level, highlighting the importance of understanding local epidemiology to adapt recommendations for initial empirical treatment. This argument is strengthened by the findings of Rabagliati et al. (2024) [29], wherein patients with ESBL (n=172, 17.2%) and KPC (n=172, 11%) bacterial isolates, mortality was higher in those who received inappropriate empirical antibiotic therapy (n=64, 41.2%) compared to those receiving appropriate therapy (n=34, 26.6%) in cases of early bacteremia (less than four days). Another possible hypothesis for the higher mortality in our study is that our patients had more comorbidities compared to those documented in local studies [6], which was identified as a factor associated with mortality by Kuderer et al. (2006) [30], who found that patients with two comorbidities had a 28.9% (n=41,779) mortality rate compared to 12.9% (n=41,799) in those with one or no underlying disease. In addition, there was a higher frequency of positive blood cultures in patients who died (59.6%, n=53 vs. 44.1%, n=192) (Table 9), similar to findings recently reported by Rabagliati et al. (2024) [29], where mortality in patients with or without documented bacteremia was 26.7% vs. 15.3%, respectively. Another important finding was the median duration of neutropenia (in days), which was longer in those who died compared to those who survived (15.5 vs. 8 days, respectively), which differs from findings by Parodi et al. (2015) (7.5 vs. 5 days) [8]. It has been described that the longer the recovery time for neutrophils (over 10 days in some series), the worse the prognosis [19].

Risk factors for mortality in FN and HM identified in other studies include advanced age, uncontrolled cancer, previous infection, body temperature ≥39°C, hypotension, dehydration, tachycardia, acute respiratory failure, septic shock, pneumonia, bacteremia, prolonged neutropenia, low platelet count, liver enzyme abnormalities, CRP, and procalcitonin [19, 31]. ICU admission, the need for vasopressor support, and mechanical ventilation have also been described as risk factors for mortality in multiple studies [3, 8, 10, 11, 18, 19, 20, 21, 23]. Unfortunately, we did not find a statistically significant relationship in the multivariate analysis for most of the mortality predictors reported in previous studies (Table 8). We speculate that factors such as insufficient variability in the data for one or more of the variables may reduce the ability to detect an association. Additionally, multicollinearity, reflected in two or more independent variables that are highly correlated (e.g., hypoalbuminemia and non-response to ceftriaxone due to albumin binding), could make it difficult to assess the individual effect of each variable. This can lead to misleading or insignificant results in regression models. However, in our study, the clinical and sociodemographic description of those who died vs. those who survived showed quantitatively higher rates of age, hypertension, immunosuppression, chronic kidney disease, chronic obstructive pulmonary disease (COPD), high-grade ECOG, high-risk MASCC (<21 points), CRP, ferritin, LDH, and lower albumin levels. Regarding some of these factors, for example, Combariza et al. (2015) [32] found that CRP greater than 15 mg/dL within the first five days of the FN episode is a risk factor for mortality in HM. Albumin levels <3.0 g/dL affect defervescence time in FN, and hypoalbuminemia is proposed as an indicator of the patient’s nutritional status, also altering the efficacy of antibiotics due to decreased protein binding [33]. In our study, mortality was related to hypoalbuminemia within the first five days of FN diagnosis, similar to the findings of Shmuely et al. (2023, 2020) [23, 34].

There are several limitations to the study, including its observational retrospective nature, which may introduce information bias due to incomplete data in medical records, potentially affecting the true impact of risk factors involved in complications and mortality. For example, LDH data were not documented for 32 (20.7%) patients at the start of FN. Additionally, we did not distinguish between early or late bacteremia, nor did we measure the time to start antibiotics when FN was documented, which has been previously described as an important risk factor for mortality [29]. We acknowledge that selection bias could affect this study since participants were selected based on existing records, and this may not represent the general population. Furthermore, those included in the study may have different characteristics compared to those who were excluded, which could affect the generalizability of the findings. On the other hand, as strengths of the study, we identify that this is one of the largest studies of FN in our setting, provides data on the clinical and microbiological aspects of FN, establishes a foundation for further research on potentially modifiable factors in patients with HM and FN, and proposes strategies that may help reduce mortality.

## Conclusions

This study provides important information on the clinical and microbiological characteristics of FN cases in our setting. We recommend routine surveillance of local microbiological resistance patterns and the development of institutional guidelines for empirical antibiotic therapy to mitigate adverse outcomes in these patients. No factors related to complications or mortality were found in the multivariate analysis. However, the heterogeneity of the population suggests that these outcomes are not determined by a single factor, and a study with a larger sample may be needed to confirm these findings.
